# A Series of Tubes: The *C. elegans* Excretory Canal Cell as a Model for Tubule Development

**DOI:** 10.3390/jdb8030017

**Published:** 2020-09-07

**Authors:** Matthew Buechner, Zhe Yang, Hikmat Al-Hashimi

**Affiliations:** 1 Department of Molecular Biosciences, University of Kansas, Lawrence, KS 66045, USA; zyang@ku.edu; 2 Deciphera, Pharmaceuticals, Inc., Lawrence, KS 66044, USA; hikmathashimi@yahoo.com

**Keywords:** epithelial tube, apical surface, vesicle transport, exocytosis, vacuolar ATPase, intermediate filaments, terminal web, IRG protein

## Abstract

Formation and regulation of properly sized epithelial tubes is essential for multicellular life. The excretory canal cell of *C. elegans* provides a powerful model for investigating the integration of the cytoskeleton, intracellular transport, and organismal physiology to regulate the developmental processes of tube extension, lumen formation, and lumen diameter regulation in a narrow single cell. Multiple studies have provided new understanding of actin and intermediate filament cytoskeletal elements, vesicle transport, and the role of vacuolar ATPase in determining tube size. Most of the genes discovered have clear homologues in humans, with implications for understanding these processes in mammalian tissues such as Schwann cells, renal tubules, and brain vasculature. The results of several new genetic screens are described that provide a host of new targets for future studies in this informative structure.

## 1. Introduction

Of the many forms of epithelial tissues that make up the bodies of living organisms, tubes may be the most complicated to build. From the short tubular shapes of secretory glands to the wide and long gastrointestinal tract that reaches the whole length of animals, tubes must grow to connect destinations often far apart, while remaining flexible enough to bend as the organism moves. In addition, the contents of the lumen (center) may have osmolarity very different from that of the epithelial cytoplasm, exerting strong pressures on the apical membrane surrounding the lumen. Finally, tubes must regulate their lumen diameter to regulate proper flow of air or liquid; too narrow and the tube becomes blocked, while being too wide often results in pressure insufficient to move material through the tube. In mammals, the function of lung sacs, kidney nephrons, glial cells, as well as secretory organs such as the liver, pancreas, and breast depend on proper regulation of tube growth during embryogenesis and subsequent growth to adulthood [1].

For larger biological tubes, the length and diameter are often determined by the number of cells along the length or within a cross-section. Frequency and direction of mitosis help determine the size of multicellular tubes. The curvature of the luminal surface is concave, opposite to the typical curvature exerted by osmotic pressure from the cytoplasm. The most drastic curvature will be found in cells with the narrowest lumen; therefore tubes where a cross-section is made up of a single cell provide especially strong models for understanding how luminal surface is curved. Understanding the growth and development of tubes with a single-celled diameter is therefore essential for understanding the development of many tissues from protozoa and plants to vertebrates.

**Figure 1:**
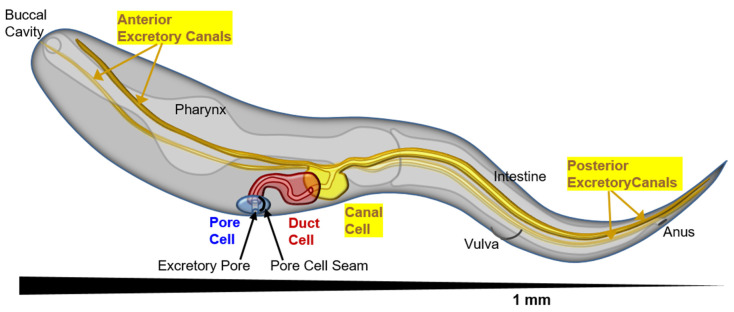
Perspective diagram (anterior close, posterior far) showing position of the cells of the excretory system, and the long tubular canals stretching the length of the nematode (1 mm total length, about 50 μm in diameter). The excretory pore cell (seamed) is in blue, excretory duct cell (seam present at birth, then removed to become seamless) in red, and excretory canal cell in yellow. The canals can collect excess liquid from the entire length of the animal to transport to the duct and pore cells for removal.

Several models of single-cell tube development have been studied historically, notably the *Paramecium* water vacuole [2], the tip cells of the *Drosophila* trachea [3,4,5], flowering plant pollen tubes [6], *Ciona* notochord [7], and narrow capillary cells in the zebrafish *Danio rerio* [8,9,10] and mammalian brain and kidney glomerulus [11,12]. The roundworm *C. elegans* offers several excellent models for understanding single-cell tubular development and growth in the nematode excretory–secretory system [13,14,15]. Here, several single-celled tubes line up with each other to form a functioning tissue that regulates organismal osmolarity and secretes various chemicals under highly disparate environments (Figure 1). Intriguingly, the three central osmoregulatory cells are formed from three different mechanisms: (a) a pore cell at the surface of the animal undergoes cell wrapping to form a junction from one side of the cell to the other; (b) a duct cell also undergoes cell wrapping, but then dissolves the junction (“autofusion”) to form a “seamless” tube with junctions only to the pore cell and to the third cell; (c) the excretory cell forms long tubes via “cell hollowing”—fusion of vesicles to form four long narrow seamless tubular “canals” stretching from the duct cell throughout the length of the animal. This arrangement allows nematodes to collect excess water from throughout the body of the organism and bring it to the duct, which regulates fluid flow (by a poorly understood mechanism) to be expelled through the pore. An excellent recent review of the range of seamless tubes in *C. elegans* was recently published by Sundaram and Cohen [15].

The excretory cell is strongly implied to regulate osmolarity, through observation of rhythmic swellings and contractions of the duct cell in conditions where the animal is not compressed (under slide covers held up by small sephadex beads) [16]. These rhythmic pulsations are inversely correlated to osmolarity of the medium, and are most evident in stages such as dauer larvae where liquid is not also passing through the gut.

Increasing interest in the excretory system has made these cells a popular subject in recent reviews [14,15,17,18,19], so this article will focus on recent genetic advances in understanding development specifically of the long excretory canals. In particular, several recent genetic screens [20,21,22] have greatly increased our knowledge of the number of molecules that act in concert to extend the long canals while maintaining a narrow, functional lumen.

## 2. Anatomy of the Canal Cell

The excretory canal cell is born on the ventral side near the pharynx about midway through embryogenesis [23]. Its sister cell goes on to become a neuron, and though the canal cell is an epithelial cell, it does express some neural characteristics, such as expression of the human Hu/*Drosophila* ELAV (Embryonic Lethal Abnormal Vision) homologue EXC-7 [24]. Very soon after the cell is born, it begins to extend dorsolaterally both left and right while vesicles coalesce to form the inner apical/luminal membrane. Upon reaching the lateral side, the left and right projections branch and continue to extend to the anterior and posterior ends of the animal, giving the cell a characteristic “H” shape. The posterior extensions only reach about halfway (near the vulva) by the time of hatching, and are not fully extended until about the middle of the first larval stage (L1), about 8 h after hatching.

The tips of the canals are closed, so each canal lumen is a dead-end; the only opening connects the canal cell to the duct cell. The canals are well-placed to collect excess liquids from across the animal. They stretch over the whole length of the animal, and are located next to the pseudocoelomic body cavity. Electron micrographs show that the canal basal surface shares a basement membrane with the hypodermal epithelium, and the canals are connected to the hypodermis by extensive gap junctions [13]. Excess liquid that leaks into the animal from the gut or the hypodermis can therefore hypothetically be pumped into the canal cytoplasm and lumen to be expelled through the duct and pore.

Strict control over canal luminal diameter is strongly implied by the size of the cell lumen, which is only 1–5 μm in diameter over the length of the canals [13]. The anterior canals are noticeably thinner than the posterior canals, and the lumen tapers as it reaches the tip of both anterior and posterior canals. The canals are also surprisingly flexible—each canal retains its long shape and luminal diameter as the animal undulates sinusoidally during movement.

**Figure 2:**
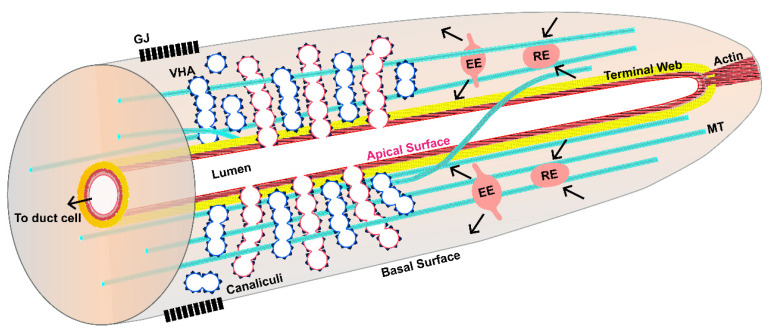
Diagram of canal section, showing subcellular morphology of lumen and canal tip. The apical membrane is shown in red surrounding the central lumen (in white), while the basal membrane is shown in grey. The apical surface is coated by actin filaments (thick red) and intermediate filaments (thick yellow) that together form the terminal web. Filaments extend to the distal terminus of the canal, presumably to help in canal extension. Small canalicular vesicles appear as separate vesicles (in blue) or connected to the lumen (in red) to form canaliculi. These vesicles are coated with vacuolar ATPase (VHA, black spikes on vesicles). Microtubules (cyan) extend along the length of the canal, interspersed with and outside the canaliculi. Some microtubules appear helically wound around the lumen. Early endosomes (EE) and recycling endosomes (RE) move along the canal length (See Supplementary Video S1). Gap junctions (black) connect the canal cytoplasm to neighboring hypoderm.

Confocal and electron micrographic studies (Figure 2) show that the canals contain many structures common to other long tubes. The central lumen is surrounded by a thick electron-dense terminal web [25] (also seen in the nematode and mammalian intestine, among many other tissues) composed of actin filaments and intermediate filaments. Actin is held to the apical membrane by the Ezrin-Radixin-Moesin homologue ERM-1 [26], which itself is activated via phosphorylation of a terminal domain upon binding to phosphatidyl inositol 4,5-bisphosphate (PIP_2_) lipid on the apical (luminal) surface [27]. Overlapping and surrounding the actin cytoskeleton is a thick layer of three intermediate filament proteins, IFA-4, IFB-1, and EXC-2/IFC-2 [22,28]. Spacing of intermediate filaments at the surface is likely mediated by ERM-1 and by the β_H_-spectrin SMA-1, which also interacts with actin filaments at the apical surface [22,29,30,31].

Most strikingly, the cytoplasm of the canals is filled with myriad small vesicles of near-uniform size (app. 50–100 nm diameter), which are visible both as separated individual endosomes, and also attached in chains to form visible canaliculi continuous with the lumen. Results from the Labouesse and Nance laboratories [32,33] have shown that the vesicles brush each other (“kiss”) to connect the lumens via small pores that allow ions and other small molecules to pass into the lumen of the canals. The vesicles are loaded with vacuolar ATPase [34]; at high magnification, the traditional V-ATPase “lollipop” structure can be seen surrounding these canalicular vesicles. The canalicular vesicles also include aquaporin [35] and (presumably) ion channels. V-ATPase acidifies vesicles [36]; when docked to the lumen, they will lower the pH of the canal lumen as well. Similarly, vacuolar ATPase is used by the *Paramecium* water vacuole to drive excess water from that organism [2]. Aquaporin permits movement of water into the vesicles, to be passed into the lumen of the canals to be excreted. Proton movement into the canal lumen presumably attracts anions. A possible candidate anion is chloride, since the intracellular chloride channel (CLIC channel) EXC-4 is essential for canal formation [37]. There are conflicting results as to the ability of CLIC to act as a channel, however [38]. Many other ion transporters have also been found to be necessary for canal morphology [14].

Surrounding the lumen, terminal web, and canaliculi are found the typical components of cells, including cytoskeleton, mitochondria, endoplasmic reticulum, Golgi, and vesicles. Electron micrographs show multiple microtubules in this region running along the anterior–posterior length of the canals. Other vesicles in this region are also evident, and fluorescence images show that larger endosomes labeled with fluorescently marked Rab proteins move along the length of the canals in healthy animals [39] (Supplemental Video S1).

Finally, at the basolateral surface gap junctions connect the canals to the hypodermis. Two innexins (invertebrate connexins), INX-12 and INX-13, are expressed at high levels primarily within the canals, and are essential for canal formation [20].

## 3. Development of the Excretory Canals

### 3.1. Outgrowth

Outgrowth of the developing canals is led by an actin-rich structure similar in appearance to a growth cone in neurons [22,24,40]. In electron micrographs, the canal appears embedded in the hypodermis [13,41], with a common basement membrane facing the pseudocoelom. It is unknown as to whether the canal secretes its own basement membrane versus insinuating itself between the hypodermal cells and their basement membrane. Guidance of canal growth utilizes many neural guidance signals. In mutants defective in netrin UNC-6 or the netrin receptor UNC-5, the two posterior canals grow next to each other along the ventral surface rather than being guided separately to the lateral surfaces [42]. Other mutants cause the canals to extend only partway to their destination near the posterior end of the animal. In particular, the protein UNC-53 (vertebrate NAV2) interacts with multiple proteins involved with extension of the actin cytoskeleton [43,44,45].

Initial formation of the luminal surface is believed to occur where the canal cell connects to the neighboring duct cell and may depend on expression of ERM-1/ezrin and EXC-4/CLIC channels [35,37]. *C. elegans* mutants lacking EXC-4 exhibit very large cysts along shortened canals. In mammalian cells, CLIC proteins are necessary for formation of the Apical Membrane Insertion Site (AMIS) that nucleates formation of apical surfaces in MDCK cells [46]. EXC-4 is targeted to the canal apical membrane via an N-terminal amphipathic helix [47]. Unlike most CLIC channels, EXC-4 (and mammalian CLIC3) does not have a nuclear localization signal [48]. In the *C. elegans* intestinal cells, a homologous protein EXL-1 is required for normal luminal morphogenesis instead of EXC-4 [48].

Developmental growth of the canals is rapid during late embryogenesis through the first half of the first larval stage [24], as the canal tips (especially the posterior tips) must catch up to the rapidly lengthening tail end of the animal. During this period, small evenly spaced swellings of the cytoplasm (called “beads” or “pearls”) are visible throughout the canals [22,49]. These swellings are believed to be areas where proteins are being rapidly synthesized in order to produce cytoskeletal elements as well as membrane to stabilize and elongate the canals. The pearls are generally no longer visible after the first larval stage, but can be induced to reappear under conditions of hypoosmotic stress [33], when the canal is presumably physiologically most active to pump out water [16].

Even though the pearls shrink to normal canal diameter when the canal reaches the ends of the animal, canal development does not stop. The canals continue to elongate to match the length of the animal as it extends at least threefold during the four larval stages and into adulthood. It is unknown as to whether elongation in these stages is: (a) concentrated at the tips of the canals (as appears to be the case in the embryonic and L1 stage); (b) spread evenly throughout the canals, as might be expected for canals segments affixed to specific areas of the hypodermis; or (c) a mixture of end- and evenly-spread growth.

### 3.2. Cytoskeleton

With many of the steps of canal development and molecules described, current research focuses on how these molecules determine canal apical lumen formation and growth in step with basal outgrowth to the ends of the animal. A wide range of mutants affecting luminal structure affords hints to these processes. In these mutants, the canals are generally shorter than normal, and in many the canal lumen swells into a series of fluid-filled cysts [41]. The size of the cysts varies from mutant to mutant, but in the most severe cases these canals exhibit almost no outgrowth, and the cysts can be larger than half the diameter of the animal. Cyst formation can be observed in embryos, and can take place nearly instantaneously [39]. The cystic phenotype, and especially the sudden appearance of cysts during embryonic development, implies that fluid pressure pushes the luminal membrane outward against a restraining force, presumably the apical cytoskeleton, which can fail stochastically in different locations. In several mutants, cysts form in embryogenesis most frequently at the cell body and at the growing tips of the canals; these observations suggest that the fastest apical growth (where the cytoskeleton is presumably still being polymerized) occurs at these areas of the canal.

**Figure 3:**
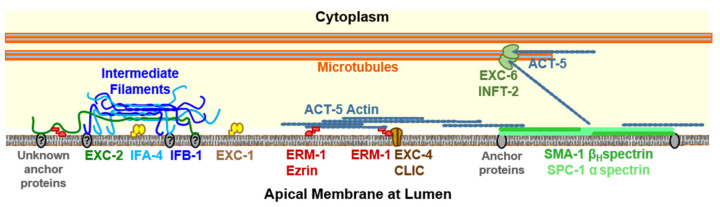
Diagram (not to scale) of cytoskeletal elements at the excretory canal apical membrane. Microtubules are located both near and far from the apical membrane. Note that not all locations and interactions have been proven to occur within the excretory canals of *C. elegans*. Tips of microtubules are located at organizing centers, where formin EXC-6 (and possibly formin INFT-2) nucleate actin filaments both along the length of the canal and towards its apical surface [55]. Fibers of ACT-5 actin are held close to the apical surface and organized there through interactions with the β-heavy spectrin SMA-1 [30,31,56] (presumably anchored by the Band 4.1 homologue FRM-1 [57]) and with the ezrin/radixin/moesin homologue ERM-1 [22,29,58]. In mammals, intracellular chloride channel (CLIC) channels homologous to EXC-4 are also associated with ezrin [59]. Intermediate filaments are associated with the luminal membrane by unknown anchor proteins, though spacing of these filaments depends on ERM-1 and SMA-1 [22,29]. The intermediate filaments associate by their central filament domain to form heterologous filaments that extend farther from the membrane than do the bulk of the apical actin filaments [60]. EXC-1 is another protein affecting canal structure located exclusively at the apical membrane [22].

It is not completely certain whether the actin filaments or the thick layer of intermediate filaments, or both, are the major structural element preventing swelling of the lumen (Figure 3). There are eight different intermediate filament genes expressed in *C. elegans* [50,51], with multiple proteins expressed in the intestinal terminal web [52,53], while three are expressed in the canals: IFA-4, IFB-1, or EXC-2 (EXC-2 has multiple isoforms, some labeled IFC-2 in studies [51,53,54]). Loss of any one of the three intermediate filament proteins surrounding the excretory canal lumen causes formation of large fluid-filled cysts, and a substantial reduction of the number of canalicular vesicles [22,28].

Intermediate filaments (IF) are similar in structure between nematodes and mammals, with a central coiled-coil filament domain used for dimerization flanked by variable N- and C-terminal domains [60,61,62]. The N- and C- termini are hydrogen-bonded so that, in reaction to a force bound to the ends, the N- and C-terminal domains can be stretched, and pull back to their original shape when that force is released [60,63]. In the excretory canals, the three IF proteins form a meshwork wrapped around the lumen of the canals [22,28], superficially similar in appearance to the meshwork of lamin filaments wrapped around the nuclear membrane [64,65]. When one or another IF gene is mutated or genetically knocked down, the meshwork appears disrupted, with wide spaces between the thicker filaments. The appearance suggests that, like fabric with crossing threads missing, the remaining filaments bunch up into thick cords at the apical surface, allowing large cysts to bulge out between the cords. Cyst formation does not occur when intermediate filaments are overexpressed [28].

EXC-2, which has a much longer N-terminal domain than do other intermediate filaments, may be key to lumen regulation. This IF remains localized to the apical surface even when IFA-4 and IFB-1 are missing [28], and new results show that its C-terminus retains the vesicle trafficking regulator EXC-9 (see below) to the apical surface as well [29].

Other cytoskeletal molecules affect both the lumen shape and the actin cytoskeleton. Loss of the luminal actin anchor ERM-1 causes similar dramatic cyst formation [26,35]. Unlike IF expression, however, overexpression of *erm-1* not only prevents cyst formation, but also strongly limits canal outgrowth. A recent genetic interaction screen [22] found that all three canal IF genes show genetic interactions with *erm-1*, so the ERM-1 protein may be anchoring both actin and intermediate filaments. In that study, genetic effects of the knockdown of tubulin protein *tbb-2* also affected location of intermediate filaments within the canals, and fluorescence images in that study also showed microtubules wound helically around the terminal web, and effects of microtubule knockdown on location of intermediate filament proteins within the canals.

Loss of the β-heavy spectrin SMA-1, which along with ERM-1 also regulates actin position [30,31], causes a unique phenotype: The canal swells but in a largely uniform manner, with few of the apparent septations between cysts characteristic of cytoskeletal failure. In addition, the wide lumen of *sma-1* mutants contains myriad canaliculi, with many more vesicles for each canaliculus than is seen in wild-type canals [41], unlike the case in other cystic canals that lack most canaliculi.

Mutants of the *exc-6* gene, encoding a homologue of vertebrate formin INF2 [40], exhibit variable but generally narrow canals, with anterior–posteriorward guidance disrupted, such that the lumen can split into two separate canals on occasion [41]. EXC-6 connects actin to microtubules along the length of the canals, mostly near the basolateral side and especially at the growing canal tips [40]. The implication is that the actin cytoskeleton is used to guide the outgrowth of growing canals during embryonic and larval development.

Two other formin proteins stimulate polymerization of actin at the apical surface downstream of the activity of the small GTPase CDC-42 [55]. INFT-2 is a second *C. elegans* INF2 homologue, while CYK-1 is homologous to vertebrate mDia and *Drosophila* Diaphanous proteins. INFT-2 promotes canal growth at the apical surface. Interestingly, CYK-1 activity appears to regulate the activity of INFT-2, as loss of activity of these formins has opposing effects on canal outgrowth and actin accumulation within the canals, and loss of CYK-1 results in higher levels of INFT-2 protein within the canals [55].

Finally, microtubules wind around the cytoplasm along the length of the canals [22], some close to the terminal web and many outside the canaliculi (Figure 2). Besides interacting with EXC-6, microtubules are the likely transport pathways of endosomes that can be seen moving from the cell body to the tips and back again. Cell-specific knockdown of tubulin proteins specifically within the canals has not been tested, however.

### 3.3. Transport

Other factors besides the cytoskeleton are important for canal growth. As noted above, the canal lumen appears to exert pressure on the luminal membrane, opposed by the apical cytoskeleton. The length of the canaliculi increases during development. In electron micrographs of L1 larva (especially the smaller anterior canals), the number of canalicular vesicles visible in a single canaliculus is often only one [41]. In a mature adult, the number of canalicular vesicles/canaliculus in the main body of the posterior canals increases to about five [33]. Though the pressure within the canals has not been proven, the presence of vacuolar ATPase and aquaporin in the canalicular vesicles suggests that the addition of more vesicles to each canaliculus drives more water into the lumen at that point. The balance between liquid driven into the lumen and constriction from intermediate filaments would naturally lead to a narrower lumen at the tips of canals, and wider lumen as the canals collect more liquid towards the cell body.

Exocytosis is a critical component of building the luminal surface of the canals. The Nance lab found that the Ras-related GTPase RAL-1 (which acts through the exocyst [66,67,68]) is collocated with apical Par proteins PAR-3 and PAR-6, and protein kinase C (PKC-1). All of these proteins are necessary for fusion of the canalicular vesicles to the luminal surface in the excretory canals [32]. The exocyst complex mediates vesicle fusion from Golgi to apical surfaces during polarized development [68,69,70]. Loss of expression of *ral-1* or of exocyst component gene *sec-5* caused formation of medium-sized cysts throughout shortened canals [32]. In electron micrographs, the canalicular vesicles that surround the lumen are largely missing, and the lumen contains fibrous electron-dense material. This phenotype (including the fibrous material) strongly resembles the Exc-2 mutant phenotype. Overexpression of *ral-1*, however, results in a few sites along the canal exhibiting a large cyst adjoining a normal-diameter lumen [32], rather than an excessively narrow lumen with few cysts seen via *exc-2* overexpression [28,29].

Other proteins located with the canalicular vesicles include DAF-6 and CHE-14, which are the Patched and Dispatched proteins of the Hedgehog signal transduction pathway [71], and are also expressed within nematode tubular glial cells [71]. RDY-2 is a nematode-specific tetraspan protein near the apical surface of the canal and adjacent excretory duct cell [72] and that is collocated with VHA (vacuolar ATPase) proteins [73] in the canal and glial tissues [34]. *rdy* and *vha* mutants show similar defects (*rdy-1* was found to be an allele of *vha-5*), including the rod-like lethality characteristic of complete failure of the excretory system to form or function [74].

The function of the exocyst in vesicle exocytosis is one component of the larger topic of vesicle trafficking. Canal development depends upon the transport of cargo along the length of the canals, as well as the amount of cargo trafficked or recycled to the cytoplasmic (basal) versus luminal (apical) membrane, which need to grow at equivalent rates during development. The cargoes likely include membrane-bound ion channels and cytoskeletal anchoring proteins, although no specific cargo proteins have been identified in transport vesicles as has been done for the nematode intestine [75].

Another major component of vesicle transport involved in canal formation are proteins of the STRiatin-Interacting Phosphatase And Kinase (STRIPAK) complex, which is involved in cell division, cell shape, and polarity in multiple tissues, including vasculature of the mammalian brain [76,77]. Mutations in several genes allow cells of narrow blood vessels to undergo abnormal proliferation and polarization, leading to formation of large fluid-filled cysts within the cerebral vasculature (Cerebral Cavernous Malformations, CCMs). The human CCM complex maintains vascular integrity and is composed of three proteins: CCM1/Krit1 (KRev Interaction Trapped 1), CCM2/Malcavernin, and CCM3/striatin (reviewed in [78]). CCM3/striatin also interacts with the STRIPAK complex (reviewed in [79]). The STRIPAK complex is conserved from fungi to humans, and it intersects functionally with multiple pathways, including cell growth and division (TOR (Target Of Rapamycin)pathway), the Hippo pathway, the JNK (Jun N-terminal Kinase) pathway, and insulin signaling pathway. STRIPAK consists of three subcomplexes: A dimer (via a coiled-coil domain) of striatins associates with a target membrane; the coiled domains further bind Protein Phosphatase 2A; and an adaptor protein (CCM3/PDCD10 in humans) binds to striatin at a different site to recruit Germinal Center Kinase (GCK) molecules. A variety of GCK molecules are used with different STRIPAK complexes at different sites in the cell, including nuclear membrane, and plasma membrane. In neurons and in hyphal fungi, STRIPAK is used to polymerize actin filaments via stimulation/repression of the Rho GTPases RHO1 or CDC42, depending on which Striatin-Interacting Protein (Strip1 or Strip2) is bound to striatin [79].

Research from the Derry lab [80] has investigated the role of components of the nematode striatin homologue CASH-1 and of homologues of related cerebral cavernous malformation proteins. They found that striatin has a role both in development of the excretory canal lumen [81], as well as in development of the central rachis of germ cells in the syncytial female gonad [82]. Within the excretory canals, loss of expression of the core STRIPAK components including CASH-1 (striatin), CCM-3 (human CCM3/PDCD10), GCK-1 (human GCK III), or FARL-11 (human STRIP1/2) cause canals to be shortened with small fluid-filled cysts. By use of fluorescent marker proteins, they also found that the Golgi apparatus within the canals is largely missing, and that recycling endosomes (marked by the small GTPase RAB-11.1, which mediates transfer of vesicles to the recycling endosome and plasma membrane from the Golgi [75,83,84] are decreased substantially in number. The canalicular vesicles, however, are more numerous and variable in size in these mutants, often larger than normal as compared to the canaliculi in wild-type animals. These effects appear to be mediated by STRIPAK’s known stimulation of CDC-42, as RNAi knockdown of *cdc-42*, but not of the small Rho GTPase genes *chw-1* (human RHOU) or *rho-1* (human RhoA), caused shortening of the canals similar to the effects of *ccm-3* mutation.

A nice recent paper from the Mitani laboratory has shown that mutation of a different gene, *arf-1.2* (encoding a homologue of ADP ribosylation factors involved in vesicular trafficking in humans), causes large vacuoles to appear in the beads that appear during growth of the canal [85]. They further found that loss of ARF-1.2 function prevented transport of the anion transporter SULP-8 to the plasma membrane (basal surface) of the canals, but had no effect on transport of the apical anion transporter SULP-4 to the luminal surface. Remarkably, mutation of the Rho-family GTPase gene *cdc-42* suppressed the Arf-1.2 mutant phenotype. CDC-42 specifies apical development [86] and is enriched near the luminal surface of the canals [39]. These results indicate that ARF-1.2 has an essential role in these tubes for basal-directed membrane traffic, and emphasizes that the balance of apical and basal-directed traffic is essential for regulated growth of a unicellular tube.

A final set of proteins involved in endosomal transport were cloned from genes found from the original excretory cyst screen [41], including proteins encoded by *exc-1*, *exc-5*, and *exc-9* (encoding homologues to: human IRGC (Immunity-Related GTPase family C) GTPase; Guanine Exchange Factor for CDC42; and small LIM-domain protein CRIP, respectively [87,88,89,90]). Mutants in these genes created irregular fluid-filled cysts that distend the canals. Overexpression of each of these genes also cause a common phenotype, whereby the lumen diameter is rescued to its normal diameter, but canal extension is shortened. Intriguingly, overexpression of *exc-5* causes the overexpression phenotype in *exc-1* and *exc-9* mutants, but not the reverse; *exc-1* overexpression similarly rescues *exc-9* mutant lumens, but not the reverse. Furthermore, overexpression of *exc-9* also provides a partial rescue of *exc-2* (intermediate filament protein) mutants [90]. EXC-9/CRIP binds to the N-terminal half of EXC-1 in a yeast 2-hybrid assay [88]. These results suggested a common pathway from EXC-2 via EXC-9, EXC-1, and EXC-5 to CDC-42 in order to regulate formation and maintenance of the tubule luminal surface.

The three *exc* genes were investigated further through labeling of canal endosomes [39,88] with markers adapted from studies on *C. elegans* intestinal cells [75,91]. Normally, endosomes marked by labeled early endosome antigen EEA-1, or by labeled recycling endosome proteins RAB-11.1 or RME-1, travel throughout the canal cytoplasm, presumably moving along microtubules attached to EXC-6 formin [40]. In *exc-1*, *exc-5*, and *exc-9* mutants, however, EEA-1 accumulates in the areas of large cysts, to the point where EEA-1 is substantially diminished along the rest of the canal length. These mutations have an opposite effect on RME-1, which is preferentially removed from the cystic areas of the canal. Other endosomal markers, such as early endosomal marker RAB-5, late endosomal marker RAB-7, lysosomal marker GLO-1, and Golgi marker mGRIP, appear unaffected. The conclusion was that the three EXC proteins regulate recycling of membrane proteins to the apical surface, starting either from early or recycling endosomes.

The protein identity of the three EXC proteins is intriguing. All three are conserved in all metazoan animals, and have been investigated in different human tissues:

1. CRIP1 (EXC-9), for Cysteine-Rich Intestinal Protein [92,93], is common in many vertebrate tissues (not solely the intestine) including embryonic neural tube, pronephros, and cranial ganglia [93]. This cytoplasmic protein consists of a single LIM domain followed by a short (20-amino acid) tail, but the functions of these domains is unknown.
2. IRGC (EXC-1) for Immunity-Related GTPase C, is a member of the IRG family of GTPase proteins [94] that includes the mammalian protein IRGM, involved in autophagic membrane formation used for defending against parasite infection. Unlike most vertebrate IRG proteins, mouse IRGC expression is not upregulated by interferon, and is found constitutively only in testes [95]. An implication of these homologies is that EXC-1 and IRGC could be involved in membrane bending, scission, or fusion during vesicle trafficking.
3. The FGD (EXC-5) family of six GEF (Guanine Exchange Factor) s in humans [96] activate Rho-GTPases, especially CDC42 [92,97]. Facio-Genital Dysplasia (FGD)1 is the locus of Aarskog–Scott Syndrome [98], in which multiple developmental defects occur, including hypertelorism, short nose, short broad hands, short stature, shawl scrotum, and other genitourinary abnormalities [99]. FGD4 and its rodent homologue Frabin are necessary for the proper development of the insulating Schwann Cells of the peripheral nervous system [100]. Humans with homozygous mutations of this gene suffer from Charcot-Marie-Tooth Syndrome Type 4H, in which the Schwann cells fail to lengthen concomitantly with the nervous system during puberty, resulting in loss of sensation and partial or complete limb paralysis [101]. It is interesting to note that a Schwann cell wrapped around a nerve bundle is topologically a single-cell tube.

**Figure 4:**
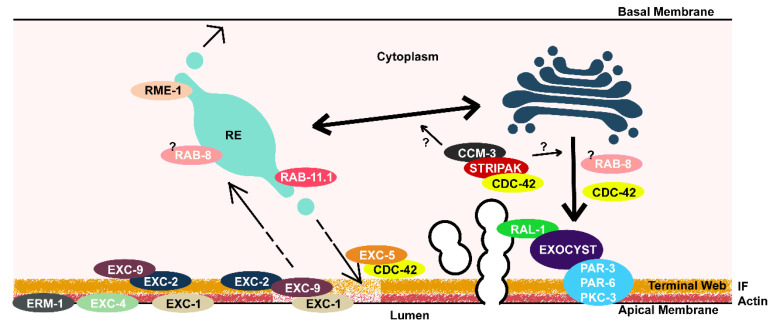
Speculative model of formation and maintenance of canal luminal shape. Lumen on bottom, basal membrane and neighboring hypodermal cell on top. Ezrin/Radixin/Moesin (ERM)-1 and EXC-1(IRG) proteins are seen at an apical membrane. EXC-2/IF and two other intermediate filaments, along with actin, make up the terminal web. The EXC-4/CLIC channel is apical. EXC-9 is retained to the apical surface by EXC-2. If terminal web is damaged or thinned during growth (light area shade of terminal web), EXC-9 can make contact with and presumably activate EXC-1. Ras domains of EXC-1 presumably trigger trafficking machinery at the recycling endosome (RE). Trafficking may be directed from the RE to apical surface, or possibly via Golgi via the STRiatin-Interacting Phosphatase And Kinase (STRIPAK) complex. EXC-5/FGD Guanine Exchange Factor activates CDC-42 to polymerize actin to bring vesicles to apical surface where the Exocyst complex and PAR complex complete fusion.

Recent results from our lab have followed up on these studies [29]. The C-terminal domain of EXC-2/intermediate filament was unexpectedly found to retain the very small (85 a.a.) LIM-domain protein EXC-9/CRIP to the terminal web, although a small fraction remained cytoplasmic. The IRGC GTPase EXC-1 was unexpectedly found almost exclusively at the apical surface, and this localization does not depend upon the presence of EXC-2 or EXC-9. Further analysis showed that EXC-1 has a predicted myristoylation domain at its N-terminus, which could help mediate accumulation at the apical surface. A final discovery found that overexpression of RAB-8, which also mediates traffic to the apical membrane in the *C. elegans* intestine, restored luminal morphology of *exc-9* and *exc-1* mutants, but not *exc-5* mutants. A model suggested from this study posed that, as the animal grows, intermediate filaments are stretched, which could allow EXC-9/CRIP to make contact with EXC-1/IRGC at the luminal surface to activate RAB-8-mediated transport which then results in EXC-5/GEF- and CDC-42-mediated actin filament polymerization and vesicle fusion with the apical surface via RAL-1 and the exocyst (Figure 4).

The new study also examined expression of trafficking proteins. RAB-11.1 and RME-1 are both found on recycling endosomes in the *C. elegans* intestine, where RAB-11.1 traffics proteins apically and RME-1 basolaterally (reviewed in [75]). In the recent work, expression in the excretory canals found that high expression of RAB-11.1 substantially diminished expression of RME-1, which suggests that the ratio of these proteins in the canals could form a natural method to balance apical and basal growth in this tube.

Finally, the interesting study by Shaye and Greenwald [40] integrated vesicle trafficking and cytoskeletal structure. Besides identifying the role of EXC-6/formin in regulating canal outgrowth, they found that EXC-6/formin and EXC-5/FGD work in parallel to regulate F-actin accumulation and canal outgrowth at the distal ends (both luminal and cytoplasmic surfaces) of growing canals.

Several outstanding questions remain to be answered concerning vesicle trafficking during canal development: (1) What is the source of canalicular vesicles? Are they formed directly from large endosomes traveling on microtubules, or are canalicular vesicles formed de novo from newly synthesized lipids from the ER or Golgi? (2) Similarly, do large endosomes recycle cargo directly from the luminal surface, from canalicular vesicles, from the basal surface, or a combination of these? (3) Perhaps most importantly, what is the identity of the cargoes carried in these endosomes?

## 4. Mutants Newly Discovered

As seen above, multiple laboratories have isolated and studied mutants that affect canal outgrowth and lumen formation and growth since the first mutants with abnormal canal morphology were observed via DIC (Differential Interference Contrast) microscopy by Edward M. Hedgecock [102]. Within the past few years, several groups have performed screens to find candidates for filling in gaps on our understanding of the mechanism of single-cell tubulogenesis in the excretory canals:

The Shaye and Greenwald labs published and revised a list of *C. elegans* genes with clear human orthologues [103,104]. The Shaye lab examined candidates by means of a focused RNAi-knockdown screen of known kinases on this list to examine formation of the excretory canals [105]. In addition to genes previously discovered used as controls for RNAi efficacy (germinal center kinases *gck-1*, *gck-3*, as well as CDC-42-binding kinase *mrck-1*, and lysine-deficient kinase *wnk-1*), they found several others, including the *mop-25.2* gene, encoding a homologue to human CAB39 Calcium-binding serine/threonine kinase, and known to interact with the dynein heavy chain [21,106]. It is therefore expected to find MOP-25.2 interacting with the series of microtubules outside the canaliculi in the developing canals.

A similar RNAi-knockdown screen from our lab [20] started with a list of genes that are highly expressed within the excretory canal cell as compared to other cells in *C. elegans* [107]. Gene knockdown via feeding-RNAi [108] was applied in strains that enhanced RNAi effects specifically within the canal. As controls, knockdown of several genes studied by others showed effects in the canals: transcription factors CEH-6 and CEH-37 described by the Bürglin, Chamberlin, and Baillie labs [109,110,111]; formin CYK-1 and kinases MOP-25.2 and GCK-3, all studied by the Shaye and Greenwald labs [55]; and the two innexins INX-12 and INX-13 that form the gap junctions of the canal basal surface to the hypodermis, as described by Hall [112]. This screen also found 12 novel genes. Some of the encoded proteins are homologous to: a Mextli regulator of translation (MXT-1 [113]); a RING finger ubiquitin ligase [114]; an F-box protein [115]; the exonuclease Egalitarian that regulates nuclear migration along dynein [116]; a SLC9 Na^+^/H^+^ channel and a Bestrophin chloride channel [117,118]; two nonspecific proteins of unknown function; and surprisingly, two proteins predicted to be involved in central metabolism, sedoheptulose kinase and an aldo-keto reductase. The function of these proteins in canal development remains to be investigated, but the results emphasize the important interplay between cytoplasmic ion content, the cytoskeleton, and vesicle trafficking required to build a tubular tissue.

Most intriguingly, the screen unexpectedly yielded two genes that, when knocked down, suppressed the effects of loss-of-function mutation (deletion of promoter and half the coding region [39]) encoding the EXC-5/FGD guanine exchange factor. Guanine exchange factors activate specific Rho-GTPases to cycle from inactive GDP-bound to active GTP-bound states, so it is surprising that loss of another protein’s function would allow the canals to grow to near-normal levels without this activation. One of these “*suex*” (SUppressor of EXc) genes (*suex-1*) encodes a novel small glycine-rich nematode-specific protein with no obvious homologues in other phyla. The other (*suex-2*) encodes a member of the SLC22 SoLute Carrier family, whose knockdown presumably alters the chemical composition of the canal cytoplasm. SLC proteins are widely expressed in the mammalian kidney and liver, both highly tubulated tissues, and suggests that alteration of these proteins in mammals could also influence FGD activity in mammals.

Perhaps the most ambitious gene screen was carried out by the Derry Lab, which has extended their work on CCM-3 through a reverse genetic screen of virtually all *C. elegans* genes looking for synthetic debilitation or lethality with the *C. elegans kri-1* gene (homologue of human CCM1/Krit1) [21]. Of the 29 genes whose knockdown caused synthetic lethality with *kri-1* homozygous mutation, 14 were found to exhibit direct effects on excretory canal development when knocked down in a wild-type background. Two hits were for genes encoding exocyst components SEC-3 (human EXOC1) and SEC-15 (EXOC6), as might be expected if the CCM and STRIPAK complexes regulate the functions of the exocyst for fusing canalicular vesicles to the lumen and to each other for passage of water into the canals [32,33]. Another hit was for *cdc-42*, which encodes the substrate of the EXC-5 guanine exchange factor [55]. A large number of other genes they found encode proteins involved in various steps of nuclear function and gene expression, including CDC-25.1 (human CDC25A) and SCC-1 (human RAD21), a cohesin complex member involved in spindle formation, transcription factors ICD-1 (BTF3) and NHR-69 (HNF4A), poly(A)-binding protein PAB-1 (PABPC4), nuclear RNA-binding protein H28G03.1 (HNRNPA1), and chaperone CCT-2 (CCT2). As the largest cell in *C. elegans*, the excretory canal cell likely requires high rates of synthesis of many canal-specific genes, so knockdown of such genes might be expected to affect canal development.

The Derry lab screen also yielded *rab-5*, encoding the small GTPase that binds to Early Endosome Antigen 1 (EEA1) to bring vesicles to the early endosome, as shown in many worm tissues, and which builds up substantially in cystic areas in many canal mutants [28,39,75,88,119,120,121]. A hit was also found for the well-known signaling protein GLP-2 (Notch), and for the gene *ephx-1* (human ARHGEF16), which encodes a Rho GEF with possible functions in migration of glioma cells [122].

Finally, this screen turned up, yet again, a strong hit on *mop-25.2*, knockdown of which caused stronger effects on the canal than did knockdown of either *ccm-3* or *gck-1* [21]. These researchers followed up the results for this gene to find that loss of MOP-25.2 function had similar effects on Golgi stability and subcellular location of RAB-11.1-labelled endosomes as did knockdown of *ccm-3* or STRIPAK components. Furthermore, *mop-25.2* expression was required to provide correct location of CCM-3 and GCK-1 to the luminal membrane of both the canals and of the multinucleate tube of the germline rachis. This relationship was also found in HUVECs (Human Umbilical Vein Endothelial Cells) in culture, indicating that the functions of these proteins has been conserved in multicellular tubular cells from nematodes to humans.

## 5. Prospects and Questions

Research on the excretory canals is creating an intriguing model for development (Figure 2, Figure 3 and Figure 4) of unicellular tubules, suggested from these observations from multiple laboratories. Outgrowth of the canals requires addition of lipids sufficient to create the long membranous luminal surface and the myriad canaliculi that surround the lumen. The canaliculi themselves supply the vacuolar ATPase and ion channels to create the osmotic gradient that concentrates liquid within the canals, which is the driving force for the expansion of the lumen. Intermediate filaments form a flexible membranous “luminal corset” that surrounds the lumen and maintains its diameter while still allowing the canals to bend as the animal moves. The diameter of the canal lumen is determined by the thickness of the terminal web squeezing the luminal membrane opposing osmotic swelling driven by the number of canalicular vesicles pushing water into the lumen. The luminal corset can stretch to expand luminal diameter as the animal goes through larval development and adds more canaliculi surrounding the lumen. These opposing forces also self-regulate the diameter of the lumen, such that the shorter anterior canals, with less water entering, will naturally form a narrower lumen than the longer posterior canals. Similarly, the tips of the canals will not be as wide in diameter as the canal at the cell body.

Formation of the canaliculi depend on movement of endosomes along microtubules surrounding the lumen, and exocytotic pathways in combination with activity of the CCM and STRIPAK complexes, as well as the EXC-9/EXC-1/EXC-5/CDC-42 (CRIP/IRG/FGD/CDC42) pathway of vesicle recycling. The discovery of a multitude of genes that cause similar canal phenotypes both enhances our knowledge of these processes and provides entrees into understanding other cellular activities required for canal development, such as lipid metabolism and links to central metabolic processes. Finally, the large degree of conservation between the genes involved in canal morphogenesis and genes in other creatures, including mammals, suggests that the mechanisms needed to form the single-celled excretory canals have been reused in the morphogenesis of many other tubules and other complicated tissue shapes throughout the range of eukaryotic life. It will be exciting to see how newly discovered factors in this single-celled tissue interact to regulate tubule development in *C. elegans*, and potentially in a wide range of other single-celled tubules throughout biology.

## Supplementary Materials

The following is available online at https://www.mdpi.com/2221-3759/8/3/17/s1, Video S1: Movement of recycling endosomes within the canal. Expression of Rab-11.1 linked to mKate2 labels recycling endosomes. Video shows movement over course of 6.3 min (compressed). The dark area in the center is the lumen in this section of canal. Canal diameter ~10 μm in diameter. Non-motile endosomes and endosomes presumably being transported along helically arrayed microtubules are evident.

## References

[1] Lubarsky B., Krasnow M.A. (2003). Tube morphogenesis: Making and shaping biological tubes. Cell.

[2] Plattner H. (2015). The contractile vacuole complex of protists—new cues to function and biogenesis. Crit. Rev. Microbiol..

[3] Maruyama R., Andrew D.J. (2012). Drosophila as a model for epithelial tube formation. Dev. Dyn..

[4] Rosa J.B., Metzstein M.M., Ghabrial A.S. (2018). An Ichor-dependent apical extracellular matrix regulates seamless tube shape and integrity. PLoS Genet..

[5] Samakovlis C., Hacohen N., Manning G., Sutherland D.C., Guillemin K., Krasnow M.A. (1996). Development of the Drosophila tracheal system occurs by a series of morphologically distinct but genetically coupled branching events. Development.

[6] Geitmann A. (2010). How to shape a cylinder: Pollen tube as a model system for the generation of complex cellular geometry. Sex. Plant Reprod..

[7] Denker E., Bocina I., Jiang D. (2013). Tubulogenesis in a simple cell cord requires the formation of bi-apical cells through two discrete PAR domains. Development.

[8] Lenard A., Daetwyler S., Betz C., Ellertsdottir E., Belting H.G., Huisken J., Affolter M. (2015). Endothelial cell self-fusion during vascular pruning. PLoS Biol..

[9] Kotini M.P., Mäe M.A., Belting H.G., Betsholtz C., Affolter M. (2019). Sprouting and anastomosis in the Drosophila trachea and the vertebrate vasculature: Similarities and differences in cell behaviour. Vascul. Pharmacol..

[10] Yu J.A., Castranova D., Pham V.N., Weinstein B.M. (2015). Single-cell analysis of endothelial morphogenesis in vivo. Development.

[11] Bär T., Güldner F.H., Wolff J.R. (1984). “Seamless” endothelial cells of blood capillaries. Cell Tissue Res..

[12] Wolff J.R., Bär T. (1972). ‘Seamless’ endothelia in brain capillaries during development of the rat’s cerebral cortex. Brain Res..

[13] Nelson F.K., Albert P.S., Riddle D.S. (1983). Fine structure of the *Caenorhabditis elegans* secretory-excretory system. J. Ultrastruct. Res..

[14] Sundaram M.V., Buechner M. (2016). The Caenorhabditis elegans excretory system: A model for tubulogenesis, cell fate specification, and plasticity. Genetics.

[15] Sundaram M.V., Cohen J.D. (2017). Time to make the doughnuts: Building and shaping seamless tubes. Semin. Cell Dev. Biol..

[16] Nelson F.K., Riddle D.L. (1984). Functional study of the Caenorhabditis elegans secretory-excretory system using laser microsurgery. J. Exp. Zool..

[17] Ganner A., Neumann-Haefelin E. (2017). Genetic kidney diseases: Caenorhabditis elegans as model system. Cell Tissue Res..

[18] Zhang N., Membreno E., Raj S., Zhang H., Khan L.A., Gobel V. (2017). The *C. elegans* excretory canal as a model for intracellular lumen morphogenesis and in vivo polarized membrane biogenesis in a single cell: Labeling by gfp-fusions, rnai interaction screen and imaging. J. Vis. Exp..

[19] Jewett C.E., Prekeris R. (2018). Insane in the apical membrane: Trafficking events mediating apicobasal epithelial polarity during tube morphogenesis. Traffic.

[20] Al-Hashimi H., Chiarelli T., Lundquist E.A., Buechner M. (2019). Novel exc genes involved in formation of the tubular excretory canals of Caenorhabditis elegans. G3 (Bethesda).

[21] Lant B., Pal S., Chapman E.M., Yu B., Witvliet D., Choi S., Zhao L., Albiges-Rizo C., Faurobert E., Derry W.B. (2018). Interrogating the CCM-3 gene network. Cell Rep..

[22] Khan L.A., Jafari G., Zhang N., Membreno E., Yan S., Zhang H., Gobel V. (2019). A tensile trilayered cytoskeletal endotube drives capillary-like lumenogenesis. J. Cell Biol..

[23] Sulston J.E., Schierenberg E., White J.G., Thomson J.N. (1983). The embryonic cell lineage of the nematode Caenorhabditis elegans. Dev. Biol..

[24] Fujita M., Hawkinson D., King K.V., Hall D.H., Sakamoto H., Buechner M. (2003). The role of the ELAV homologue EXC-7 in the development of the Caenorhabditis elegans excretory canals. Dev. Biol..

[25] Sauer F.C. (1937). Some factors in the morphogenesis of vertebrate embryonic epithelia. J. Morphol..

[26] Göbel V., Barrett P.L., Hall D.H., Fleming J.T. (2004). Lumen morphogenesis in *C. elegans* requires the membrane-cytoskeleton linker ERM-1. Dev. Cell.

[27] Ramalho J.J., Sepers J.J., Nicolle O., Schmidt R., Cravo J., Michaux G., Boxem M. (2020). C-terminal phosphorylation modulates ERM-1 localization and dynamics to control cortical actin organization and support lumen formation during Caenorhabditis elegans development. Development.

[28] Al-Hashimi H., Hall D.H., Ackley B.D., Lundquist E.A., Buechner M. (2018). Tubular excretory canal structure depends on intermediate filaments EXC-2 and IFA-4 in caenorhabditis elegans. Genetics.

[29] Yang Z., Mattingly B.C., Hall D.H., Ackley B.D., Buechner M. (2020). Terminal web and vesicle trafficking proteins mediate nematode single-cell tubulogenesis. J. Cell Biol..

[30] McKeown C., Praitis V., Austin J. (1998). *Sma-1* encodes a β_H_-spectrin homolog required for *Caenorhabditis elegans* morphogenesis. Development.

[31] Praitis V., Ciccone E., Austin J. (2005). SMA-1 spectrin has essential roles in epithelial cell sheet morphogenesis in *C. elegans*. Dev. Biol..

[32] Armenti S.T., Chan E., Nance J. (2014). Polarized exocyst-mediated vesicle fusion directs intracellular lumenogenesis within the *C. elegans* excretory cell. Dev. Biol..

[33] Kolotuev I., Hyenne V., Schwab Y., Rodriguez D., Labouesse M. (2013). A pathway for unicellular tube extension depending on the lymphatic vessel determinant PROX1 and on osmoregulation. Nat. Cell Biol..

[34] Liegeois S., Benedetto A., Garnier J.M., Schwab Y., Labouesse M. (2006). The V0-ATPase mediates apical secretion of exosomes containing hedgehog-related proteins in Caenorhabditis elegans. J. Cell Biol..

[35] Khan L.A., Zhang H., Abraham N., Sun L., Fleming J.T., Buechner M., Hall D.H., Gobel V. (2013). Intracellular lumen extension requires ERM-1-dependent apical membrane expansion and AQP-8-mediated flux. Nat. Cell Biol..

[36] Cotter K., Stransky L., McGuire C., Forgac M. (2015). Recent insights into the structure, regulation, and function of the V-ATPases. Trends Biochem. Sci..

[37] Berry K.L., Bulow H.E., Hall D.H., Hobert O. (2003). A *C. elegans* CLIC-like protein required for intracellular tube formation and maintenance. Science.

[38] Argenzio E., Moolenaar W.H. (2016). Emerging biological roles of Cl^-^ intracellular channel proteins. J. Cell Sci..

[39] Mattingly B.C., Buechner M. (2011). The FGD homologue EXC-5 regulates apical trafficking in *C. elegans* tubules. Dev. Biol..

[40] Shaye D.D., Greenwald I. (2015). The disease-associated formin INF2/EXC-6 organizes lumen and cell outgrowth during tubulogenesis by regulating F-actin and microtubule cytoskeletons. Dev. Cell.

[41] Buechner M., Hall D.H., Bhatt H., Hedgecock E.M. (1999). Cystic canal mutants in *Caenorhabditis elegans* are defective in the apical membrane domain of the renal (excretory) cell. Dev. Biol..

[42] Hedgecock E.M., Culotti J.G., Hall D.H. (1990). The *unc-5*, *unc-6*, and *unc-40* genes guide circumferential migrations of pioneer axons and mesodermal cells on the epidermis in *C. elegans*. Neuron.

[43] Stringham E., Pujol N., Vandekerckhove J., Bogaert T. (2002). UNC-53 controls longitudinal migration in *C. eegans*. Development.

[44] Bhat J.M., Pan J., Hutter H. (2015). Plr-1, a putative E3 ubiquitin ligase, controls cell polarity and axonal extensions in *C. elegans*. Dev. Biol..

[45] Wang Z., Chi Q., Sherwood D.R. (2014). Mig-10 (lamellipodin) has netrin-independent functions and is a FOS-1A transcriptional target during anchor cell invasion in *C. elegans*. Development.

[46] Chou S.Y., Hsu K.S., Otsu W., Hsu Y.C., Luo Y.C., Yeh C., Shehab S.S., Chen J., Shieh V., He G.A. (2016). CLIC4 regulates apical exocytosis and renal tube luminogenesis through retromer- and actin-mediated endocytic trafficking. Nat. Commun..

[47] Berry K.L., Hobert O. (2006). Mapping functional domains of chloride intracellular channel (CLIC) proteins in vivo. J. Mol. Biol..

[48] Liang J., Shaulov Y., Savage-Dunn C., Boissinot S., Hoque T. (2017). Chloride intracellular channel proteins respond to heat stress in Caenorhabditis elegans. PLoS ONE.

[49] Hahn-Windgassen A., Van Gilst M.R. (2009). The Caenorhabditis elegans HNF4α homolog, NHR-31, mediates excretory tube growth and function through coordinate regulation of the vacuolar ATPase. PLoS Genet..

[50] Dodemont H., Riemer D., Ledger N., Weber K. (1994). Eight genes and alternative RNA processing pathways generate an unexpectedly large diversity of cytoplasmic intermediate filament proteins in the nematode Caenorhabditis elegans. EMBO J..

[51] Karabinos A. (2019). Intermediate filament (IF) proteins IFA-1 and IFB-1 represent a basic heteropolymeric IF cytoskeleton of nematodes: A molecular phylogeny of nematode IFs. Gene.

[52] Coch R.A., Leube R.E. (2016). Intermediate filaments and polarization in the intestinal epithelium. Cells.

[53] Geisler F., Coch R.A., Richardson C., Goldberg M., Bevilacqua C., Prevedel R., Leube R.E. (2020). Intestinal intermediate filament polypeptides in *C. elegans*: Common and isotype-specific contributions to intestinal ultrastructure and function. Sci. Rep..

[54] Hüsken K., Wiesenfahrt T., Abraham C., Windoffer R., Bossinger O., Leube R.E. (2008). Maintenance of the intestinal tube in Caenorhabditis elegans: The role of the intermediate filament protein IFC-2. Differentiation.

[55] Shaye D.D., Greenwald I. (2016). A network of conserved formins, regulated by the guanine exchange factor EXC-5 and the GTPase CDC-42, modulates tubulogenesis in vivo. Development.

[56] Wirshing A.C.E., Cram E.J. (2010). Spectrin regulates cell contractility through production and maintenance of actin bundles in the Caenorhabditis elegans spermatheca. Mol. Biol. Cell.

[57] Van Furden D., Johnson K., Segbert C., Bossinger O. (2004). The *C. elegans* ezrin-radixin-moesin protein ERM-1 is necessary for apical junction remodelling and tubulogenesis in the intestine. Dev. Biol..

[58] Fehon R.G., McClatchey A.I., Bretscher A. (2010). Organizing the cell cortex: The role of ERM proteins. Nat. Rev. Mol. Cell Biol..

[59] Jiang L., Phang J.M., Yu J., Harrop S.J., Sokolova A.V., Duff A.P., Wilk K.E., Alkhamici H., Breit S.N., Valenzuela S.M. (2014). CLIC proteins, ezrin, radixin, moesin and the coupling of membranes to the actin cytoskeleton: A smoking gun?. Biochim. Biophys. Acta.

[60] Herrmann H., Aebi U. (2016). Intermediate filaments: Structure and assembly. Cold Spring Harb. Perspect. Biol..

[61] Zuela N., Gruenbaum Y. (2016). Intermediate filaments in Caenorhabditis elegans. Methods Enzymol..

[62] Gruenbaum Y., Aebi U. (2014). Intermediate filaments: A dynamic network that controls cell mechanics. F1000Prime Rep..

[63] Hohmann T., Dehghani F. (2019). The cytoskeleton-a complex interacting meshwork. Cells.

[64] de Leeuw R., Gruenbaum Y., Medalia O. (2017). Nuclear lamins: Thin filaments with major functions. Trends Cell Biol..

[65] Gruenbaum Y., Foisner R. (2015). Lamins: Nuclear intermediate filament proteins with fundamental functions in nuclear mechanics and genome regulation. Annu. Rev. Biochem..

[66] Liu J., Guo W. (2012). The exocyst complex in exocytosis and cell migration. Protoplasma.

[67] Hyenne V., Apaydin A., Rodriguez D., Spiegelhalter C., Hoff-Yoessle S., Diem M., Tak S., Lefebvre O., Schwab Y., Goetz J.G. (2015). RAL-1 controls multivesicular body biogenesis and exosome secretion. J. Cell Biol..

[68] Zago G., Biondini M., Camonis J., Parrini M.C. (2019). A family affair: A Ral-exocyst-centered network links Ras, Rac, Rho signaling to control cell migration. Small GTPases.

[69] Lepore D.M., Martínez-Núñez L., Munson M. (2018). Exposing the elusive exocyst structure. Trends Biochem. Sci..

[70] Lipschutz J.H. (2019). The role of the exocyst in renal ciliogenesis, cystogenesis, tubulogenesis, and development. Kidney Res. Clin. Pract..

[71] Perens E.A., Shaham S.C. (2005). *C. elegans* daf-6 encodes a patched-related protein required for lumen formation. Dev. Cell.

[72] Gill H.K., Cohen J.D., Ayala-Figueroa J., Forman-Rubinsky R., Poggioli C., Bickard K., Parry J.M., Pu P., Hall D.H., Sundaram M.V. (2016). Integrity of narrow epithelial tubes in the *C. elegans* excretory system requires a transient luminal matrix. PLoS Genet..

[73] Oka T., Futai M. (2000). Requirement of V-ATPase for ovulation and embryogenesis in Caenorhabditis elegans. J. Biol. Chem..

[74] Liegeois S., Benedetto A., Michaux G., Belliard G., Labouesse M. (2007). Genes required for osmoregulation and apical secretion in Caenorhabditis elegans. Genetics.

[75] Sato K., Norris A., Sato M., Grant B.D. (2014). C. elegans as a model for membrane traffic. WormBook: The Online Review of C. elegans Biology [Internet].

[76] Abdelilah-Seyfried S., Tournier-Lasserve E., Derry W.B. (2020). Blocking signalopathic events to treat cerebral cavernous malformations. Trends Mol. Med..

[77] Hwang J., Pallas D.C. (2014). STRIPAK complexes: Structure, biological function, and involvement in human diseases. Int. J. Biochem. Cell Biol..

[78] Kim J. (2016). Introduction to cerebral cavernous malformation: A brief review. BMB Rep..

[79] Kück U., Radchenko D., Teichert I. (2019). STRIPAK, a highly conserved signaling complex, controls multiple eukaryotic cellular and developmental processes and is linked with human diseases. Biol. Chem..

[80] Popiel E., Derry W.B. (2020). Generation and analysis of CCM phenotypes in *C. elegans*. Methods Mol. Biol..

[81] Lant B., Yu B., Goudreault M., Holmyard D., Knight J.D., Xu P., Zhao L., Chin K., Wallace E., Zhen M. (2015). CCM-3/STRIPAK promotes seamless tube extension through endocytic recycling. Nat. Commun..

[82] Pal S., Lant B., Yu B., Tian R., Tong J., Krieger J.R., Moran M.F., Gingras A.C., Derry W.B. (2017). CCM-3 promotes *C. elegans* germline development by regulating vesicle trafficking cytokinesis and polarity. Curr. Biol..

[83] Prekeris R., Klumperman J., Scheller R.H. (2000). A Rab11/Rip11 protein complex regulates apical membrane trafficking via recycling endosomes. Mol. Cell.

[84] Zhang N., Wang X., Gobel V., Zhang X. (2018). The galectin LEC-5 is a novel binding partner for RAB-11. Biochem. Biophys. Res. Commun..

[85] Kage-Nakadai E., Sun S., Iwata S., Yoshina S., Nishikawa Y., Mitani S. (2019). The small GTPase ARF-1.2 is a regulator of unicellular tube formation in Caenorhabditis elegans. J. Physiol. Sci..

[86] Pichaud F., Walther R.F., Nunes de Almeida F. (2019). Regulation of CDC42 and its effectors in epitheliamorphogenesis. J. Cell Sci..

[87] Gao J., Estrada L., Cho S., Ellis R.E., Gorski J.L. (2001). The Caenorhabditis elegans homolog of FGD1, the human CDC42 gef gene responsible for faciogenital dysplasia, is critical for excretory cell morphogenesis. Hum. Mol. Genet..

[88] Grussendorf K.A., Trezza C.J., Salem A.T., Al-Hashimi H., Mattingly B.C., Kampmeyer D.E., Khan L.A., Hall D.H., Gobel V., Ackley B.D. (2016). Facilitation of endosomal recycling by an IRG protein homolog maintains apical tubule structure in Caenorhabditis elegans. Genetics.

[89] Suzuki N., Buechner M., Nishiwaki K., Hall D.H., Nakanishi H., Takai Y., Hisamoto N., Matsumoto K. (2001). A putative GDP-GTP exchange factor is required for development of the excretory cell in Caenorhabditis elegans. EMBO Rep..

[90] Tong X., Buechner M. (2008). CRIP homologues maintain apical cytoskeleton to regulate tubule size in *C. elegans*. Dev. Biol..

[91] Shi A., Grant B.D. (2015). In vivo analysis of recycling endosomes in Caenorhabditis elegans. Methods Cell Biol..

[92] Cousins R.J., Lanningham-Foster L. (2000). Regulation of cysteine-rich intestinal protein, a zinc finger protein, by mediators of the immune response. J. Infect. Dis..

[93] Hempel A., Kühl S.J. (2014). Comparative expression analysis of cysteine-rich intestinal protein family members CRIP1, 2 and 3 during Xenopus laevis embryogenesis. Int. J. Dev. Biol..

[94] Pilla-Moffett D., Barber M.F., Taylor G.A., Coers J. (2016). Interferon-inducible GTPases in host resistance, inflammation and disease. J. Mol. Biol..

[95] Bekpen C., Hunn J.P., Rohde C., Parvanova I., Guethlein L., Dunn D.M., Glowalla E., Leptin M., Howard J.C. (2005). The interferon-inducible p47 (IRG) gtpases in vertebrates: Loss of the cell autonomous resistance mechanism in the human lineage. Genome Biol..

[96] Eitzen G., Smithers C.C., Murray A.G., Overduin M. (2019). Structure and function of the FGD family of divergent FYVE domain proteins. Biochem. Cell Biol..

[97] Zheng Y., Fischer D.J., Santos M.F., Tigyi G., Pasteris N.G., Gorski J.L., Xu Y. (1996). The faciogenital dysplasia gene product FGD1 functions as a CDC42hs- specific guanine-nucleotide exchange factor. J. Biol. Chem..

[98] Pasteris N.G., Cadle A., Logie L.J., Porteous M.E., Schwartz C.E., Stevenson R.E., Glover T.W., Wilroy R.S., Gorski J.L. (1994). Isolation and characterization of the faciogenital dysplasia (Aarskog- Scott syndrome) gene: A putative Rho/Rac guanine nucleotide exchange factor. Cell.

[99] Orrico A., Galli L., Falciani M., Bracci M., Cavaliere M.L., Rinaldi M.M., Musacchio A., Sorrentino V. (2000). A mutation in the pleckstrin homology (PH) domain of the FGD1 gene in an Italian family with faciogenital dysplasia (Aarskog-Scott syndrome). FEBS Lett..

[100] Delague V., Jacquier A., Hamadouche T., Poitelon Y., Baudot C., Boccaccio I., Chouery E., Chaouch M., Kassouri N., Jabbour R. (2007). Mutations in FGD4 encoding the Rho GDP/GTP exchange factor Frabin cause autosomal recessive Charcot-Marie-Tooth type 4H. Am. J. Hum. Genet..

[101] Vallat J.M., Mathis S., Funalot B. (2013). The various Charcot-Marie-Tooth diseases. Curr. Opin. Neurol..

[102] Hedgecock E.M., Culotti J.G., Hall D.H., Stern B.D. (1987). Genetics of cell and axon migrations in Caenorhabditis elegans. Development.

[103] Kim W., Underwood R.S., Greenwald I., Shaye D.D. (2018). Ortholist 2: A new comparative genomic analysis of human and Caenorhabditis elegans genes. Genetics.

[104] Shaye D.D., Greenwald I. (2011). Ortholist: A compendium of *C. elegans* genes with human orthologs. PLoS ONE.

[105] Shaye D.D. (2020). Personal communication.

[106] O’Rourke S.M., Dorfman M.D., Carter J.C., Bowerman B. (2007). Dynein modifiers in *C. elegans*: Light chains suppress conditional heavy chain mutants. PLoS Genet..

[107] Spencer W.C., Zeller G., Watson J.D., Henz S.R., Watkins K.L., McWhirter R.D., Petersen S., Sreedharan V.T., Widmer C., Jo J. (2011). A spatial and temporal map of *C. elegans* gene expression. Genome Res..

[108] Kamath R.S., Fraser A.G., Dong Y., Poulin G., Durbin R., Gotta M., Kanapin A., Le Bot N., Moreno S., Sohrmann M. (2003). Systematic functional analysis of the Caenorhabditis elegans genome using RNAi. Nature.

[109] Hench J., Henriksson J., Abou-Zied A.M., Luppert M., Dethlefsen J., Mukherjee K., Tong Y.G., Tang L., Gangishetti U., Baillie D.L. (2015). The homeobox genes of Caenorhabditis elegans and insights into their spatio-temporal expression dynamics during embryogenesis. PLoS ONE.

[110] Armstrong K.R., Chamberlin H.M. (2010). Coordinate regulation of gene expression in the *C. elegans* excretory cell by the POU domain protein CEH-6. Mol. Genet. Genomics.

[111] Burglin T.R., Ruvkun G. (2001). Regulation of ectodermal and excretory function by the *C. elegans* POU homeobox gene ceh-6. Development.

[112] Hall D.H. (2017). Gap junctions in *C. elegans*: Their roles in behavior and development. Dev. Neurobiol..

[113] Peter D., Weber R., Kone C., Chung M.Y., Ebertsch L., Truffault V., Weichenrieder O., Igreja C., Izaurralde E. (2015). Mextli proteins use both canonical bipartite and novel tripartite binding modes to form eIF4E complexes that display differential sensitivity to 4E-BP regulation. Genes Dev..

[114] Kaneko M., Iwase I., Yamasaki Y., Takai T., Wu Y., Kanemoto S., Matsuhisa K., Asada R., Okuma Y., Watanabe T. (2016). Genome-wide identification and gene expression profiling of ubiquitin ligases for endoplasmic reticulum protein degradation. Sci. Rep..

[115] Kipreos E.T., Pagano M. (2000). The F-box protein family. Genome Biol..

[116] Fridolfsson H.N., Ly N., Meyerzon M., Starr D.A. (2010). UNC-83 coordinates kinesin-1 and dynein activities at the nuclear envelope during nuclear migration. Dev. Biol..

[117] Fuster D.G., Alexander R.T. (2014). Traditional and emerging roles for the SLC9 Na^+^/H^+^ exchangers. Pflugers Arch..

[118] Strauß O., Müller C., Reichhart N., Tamm E.R., Gomez N.M. (2014). The role of bestrophin-1 in intracellular Ca(^2+^) signaling. Adv. Exp. Med. Biol..

[119] Liu H., Wang S., Hang W., Gao J., Zhang W., Cheng Z., Yang C., He J., Zhou J., Chen J. (2018). LET-413/Erbin acts as a Rab-5 effector to promote Rab-10 activation during endocytic recycling. J. Cell. Biol..

[120] Law F., Seo J.H., Wang Z., DeLeon J.L., Bolis Y., Brown A., Zong W.X., Du G., Rocheleau C.E. (2017). The VPS34 PI3K negatively regulates RAB-5 during endosome maturation. J. Cell Sci..

[121] Liu O., Grant B.D. (2015). Basolateral endocytic recycling requires RAB-10 and AMPH-1 mediated recruitment of RAB-5 GAP TBC-2 to endosomes. PLoS Genet..

[122] Huang D., Wang Y., Xu L., Chen L., Cheng M., Shi W., Xiong H., Zalli D., Luo S. (2018). GLI2 promotes cell proliferation and migration through transcriptional activation of ARHGEF16 in human glioma cells. J. Exp. Clin. Cancer Res..

